# Benign Paroxysmal Positional Vertigo: comparison of two recent international guidelines

**DOI:** 10.1590/S1808-86942011000200009

**Published:** 2015-10-19

**Authors:** André Luís dos Santos Silva, Marina Reis Campos Marinho, Fabiana Maria de Vasconcelos Gouveia, Julio Guilherme Silva, Arthur de Sá Ferreira, Renato Cal

**Affiliations:** 1 PhD in Physical Therapy. Adjunct Professor of the Graduate Program in Rehabilitation Sciences - UNISUAM - Rio de Janeiro - RJ; 2 Physical Therapist - UFPE. Major interest in Vestibular Rehabilitation; 3 MSc. Professor - Department of Physical Therapy - UFPE; 4 PhD. Adjunct Professor of the Graduate Program in Rehabilitation Sciences - UNISUAM - Rio de Janeiro- RJ; 5 PhD. Adjunct Professor of the Graduate Program in Rehabilitation Sciences - UNISUAM - Rio de Janeiro - RJ; 6 ENT. Otology and Neurotology Fellow - Harvard University - USA. Preceptor at the ENT Medical Residency Program in The Federal University of Pará. Graduate Program in Rehabilitation Sciences - UNISUAM - Rio de Janeiro- RJ

**Keywords:** practice guidelines as topic, rehabilitation, vertigo

## Abstract

Benign Paroxysmal Positional Vertigo (BPPV) is characterized by vertigo, lasting for a few seconds and usually managed by head positioning maneuvers. To educate clinicians concerning the state-of-the art knowledge about its management, the international societies developed guidelines.

**Aim:**

the aim of this paper is to discuss, in a practical fashion, the current options available to manage BPPV.

**Method:**

Study design: non-systematic review. This study reviews two recent guidelines regarding the evaluation and treatment of BPPV. The first one was published by the American Academy of Otolaryngology Head and Neck surgery (AAO-HNS) and the other by the American Academy of Neurology (AAN). The similarities were presented in different tables.

**Results:**

Those guidelines presented differences regarding methods. Only the AAO-HNS guidelines recommend the Dix-Hallpike test for the diagnosis of BPPV. Only canalith repositioning maneuver, Semont maneuver and vestibular rehabilitation had showed some benefit and were recommended as good treatment options.

**Conclusions:**

Both guidelines fulfilled all the aspects required for clinicians to diagnosed and manage BPPV; only the AAO-HNS's guidelines were more comprehensive and of better quality.

## INTRODUCTION

Vertigo corresponds to the feeling of rotation in the environment or having the environment rotate around oneself^1^. Benign Paroxysmal Positional Vertigo (BPPV), described in 1921 is very likely the most common cause of vertigo, with a prevalence of 20%-30% in specialized clinics^2^, ^3^. The main symptom is the feeling of rotational dizziness triggered by a change in head position. It can happen in an unpredictable and sudden way, but it does not have a progressive pattern^4^. Parnes et al.^5^ reported that approximately 58% of the BPPV cases do not have a clearly identified cause. Its primary form corresponds to 50-70% of the cases. On the other hand, the second most common cause is head injury (7%-17%), followed by vestibular neuritis (15%). With an annual incidence of 0.6%, it affects more women than men, and its prevalence is seven times higher in people older than 60 years, with an age peak between 70 and 78 years. Consanguineous relatives have five times more likelihood of developing BPPV^6^. In a German epidemiological study, Brevern et al.^7^ reported that 86% of the interviewed individuals had important psychosocial limitations which prevented them from developing their daily activities, they avoided driving or leaving their homes during the spells, and most of them ended up developing depression and anxiety^7^. In a North-American epidemiological study, the calculated expenses to control BPPV reached the sum of two thousand dollars per patient. Most of these costs were not necessary and it was associated with misdiagnosis and inefficient treatment^8^. Another study, in England, calculated that the time between BPPV's onset until effective clinical treatment was of 92 weeks^9^. The diagnosis of this condition is based on clinical history, followed or not by vomit, instability and unbalance. Different maneuvers can be used to confirm the diagnosis. The Dix-Hallpike maneuver is the most used for the posterior and anterior canals, and it should be done by qualified professionals. Diagnostic criteria include torsional nystagmus and a feeling of vertigo. For horizontal canal BPPV, we use the *roll-test*, turning the patient's head in its own plane^10^, ^11^, ^12^, ^13^, ^14^, ^15^. Today, there are three basic treatments for BPPV, with their own indications: canalith repositioning maneuver, freeing exercises and the Brandt-Daroff habituation exercises. The choice of which maneuver or exercise is more adequate will depend on the canal involved and the type of BPPV. Usually, the canalith repositioning maneuver is used in cases of canalolithiasis or the freeing maneuver for cupulolithiasis. The habituation exercises are more used with patients with residual and milder complaints^16^, ^17^. In an attempt to better organize the ideas concerning the techniques to be used for the diagnosis and treatment of BPPV, Fife et al.^18^ and Bhattacharyya et al.^19^ created practical guidelines. The goal of the present paper is to discuss, in a practical and didactical way, the current approach available concerning evaluation and treatment for BPPV.

## MATERIALS AND METHODS

This study was an asystematic review with a critical analysis comparing two international guidelines concerning BPPV evaluation, diagnostic and treatment. We chose two papers which aimed at establishing a world consensus on the matter, both published in 2008. One was supervised by the *American Academy of Neurology* (AAN)^18^ and the other was supervised by the *American Academy of Otolaryngology* (AAO-HNS)^19^, which were published in different journals in the fields of Neurology and Otorhinolaryngology, respectively. The *AAO-HNS* formally established that the clinical practice guidelines were not conceived as the sole source of guidance in the control of BPPV. On the contrary, the intent was to provide support to clinicians as a structure based on evidence for decision making and to define management strategies. The authors explained that the paper was not intended for replacing clinical judgment or to establish a protocol to be followed for all individuals with such condition, especially because it could not provide for one single adequate approach to diagnose and control the problem. The results were presented in comparative tables and the common topics were compared and discussed in order to check the impact of the guidelines concerning each type of recommendation presented by the respective authors and/or academies.

## RESULTS

Upon checking both papers, in regards of the methodology used for each study, it was possible to identify some basic differences. While the paper published by the AAN^18^ had only neurologists and neurotologists in their team of researchers, the second, under the auspices of the AAO-HNS^19^, had a multidisciplinary team of investigators, involving not only otolaryngologists, but also other professional representatives of the following fields: physical therapy, osteopathy, emergency medicine, Family practice, geriatrics, internal medicine, neurology, head and neck surgery, audiologists, physiatrists and rehabilitation professionals. The goals were also distinct, one was broader and with the goal of improving the quality of diagnosis and treatment (AAO-HNS)^19^, while the other was dedicated to answers questions concerning only treatment (AAN)^18^(Table 1).

**Table 1:** Comparison of the goals of both International Guidelines.

Fife et al., Neurology, 2008^18^ *American Academy of Neurology (AAN)*	Bhattacharyya et al., Otolaryngology - Head and Neck Surgery, 2008^19^ *American Academy of Otolaryngology - Head and Neck Surgery Foundation (AAO - HNS)*
Answer the following questions: - Which maneuvers are able to efficiently manage posterior canal BPPV? - Which maneuvers are efficient for the anterior and horizontal canal BPPV? - Are post-maneuver restrictions necessary? - Is the simultaneous mastoid vibration important for maneuver efficacy? - How efficient are the Brandt-Daroff habituation exercises or the maneuvers self-administered by the patients? - Are medications efficient to manage BPPV? - Are the surgical occlusion of the posterior canal or singular neurectomy effective to manage BPPV?	- To improve care quality and the results concerning BPPV by improving diagnosis accuracy and efficiency, - To reduce the inadequate use of drugs which suppress vestibular function, - To reduce the inadequate use of complementary tests such as x-rays and vestibular tests, and to increase the rational use of repositioning therapeutic maneuvers, - To engage all professional who can diagnose and treat BPPV patients, - To enable its use in any setting where BPPV is identified, monitored or controlled.

The studies found by Fife et al.^18^ followed the AAN evidence system classification, dividing it into Classes I, II, III and IV, and the recommendations were made according to AAN criteria, in order to translate the quality of this evidence, and they could be classified in levels: A (efficient), B (probably efficient), C (possibly efficient) or U (insufficient data)^18^ Bhattacharyya et al.^19^ used the Statements’ Policies recommended by the *American Academy of Pediatrics Steering Committee on Quality Improvement and Management* (AAP SCQIM), according to which studies are divided into classes A, B, C, D, X, resulting in the following degrees of recommendation: “Strongly Recommended “, “Recommended”, “Optional” and “Not Recommended”. In each classification, the level of evidence and degrees of recommendation varied from the most reliable all the way to studies with less scientific evidence. (Tables 4 and 5).

**Table 4:** Comparison of the levels of evidence and degrees of recommendation of the studies selected in the Guidelines:

Fife et al., Neurology, 2008^18^ *American Academy of Neurology (AAN)*	Bhattacharyya et al., 2008^19^ Otolaryngology - Head and Neck Surgery (AAO-HNS)
Class I: Clinical-randomized, prospective, blind trial, in a representative population. Class II: Cohort, prospective study in a representative population, blinded in the assessment of the results OR a randomized, controlled clinical trial in a representative population. Class III: All other controlled trials (including well-defined controls on the natural history or ill-patients serving as their own controls) in a representative population, in which the results are independently assessed, or in an independently determined fashion by a measure of objective results. Class IV: Evidence from uncontrolled studies, case series, case reports or expert's opinion.	Class A: Well-outlined, randomized controlled studies or studies of diagnosis done in a population similar to the target population of the paper. Class B: Randomized, controlled studies or studies of diagnosis with minor limitations; hugely consistent evidences from observational studies. Class C: Observational studies (case-controlled, cohort). Class D: Expert's opinion, case reports, reasoning from experimental studies (research bank or animal studies). Class X: Special situations for which validated studies could not have been carried out should there be a clear preponderance of the benefits over the risks.

**Table 5:** Comparing the recommendations from both academies.

Fife et al., Neurology, 2008^18^ *American Academy of Neurology (AAN)*	Bhattacharyya et al., 2008^19^ Otolaryngology - Head and Neck Surgery (AAO-HNS).
Level A: Established as efficient, inefficient or harmful for a given condition on the specified population. (requiring at least two consistent class I studies.) Level B: Probably efficient, inefficient or harmful for the condition in the specified population. (Level B required at least one Class I study or at least two consistent Class II studies). Level C: Possibly efficient, inefficient, or harmful for the condition in the specified population. (Class C classification level required at least one Class II study or at least to Class III consistent studies.) Level U: Inadequate or conflicting data with the current knowledge, treatment would not be approved. · The recommendations could have been positive or negative.	“Strong recommendation”: the benefits were clearly higher than the risks and the quality of support evidence was excellent (class A or B). In some clearly identified circumstances, it could be based in less evidence when possible to obtain high quality proof and the benefits would clearly outweigh the risks. “Recommendation”: the benefits were greater than the risks, but the quality of the evidence was not so strong (class B or C). Could have been made under the same previous conditions (A or B). “Option”: the quality of the existing evidence was suspicious (class D) or well conducted studies (class A, B or C) showed small advantages of one method over the other. “Without recommendations”: there was a lack of pertaining evidence (class D), as a not very clear balance of the risks and benefits.

In regards of classes I and II studies from the *AAN*, besides the mentioned criteria; in order to be classified, the papers should have: a) a clearly defined primary result; b) Clearly defined inclusion/exclusion criteria; c) proper accounting for drop outs and cross-over studies with sufficiently low numbers to have a minimum potential of a bias; d) relevant and substantially equivalent base characteristics between treatment groups or, should there be, proper statistical adjustment for the differences.

In relation to the subjects that each topic portrayed, AAN proposed some questionings concerning the treatment of BPPV^18^. On the other hand, the findings from Bhattacharyya et al. were broken down into thirteen statements based on evidence^19^ (Table 6).

**Table 6:** Issues approached by the respective authors of the international guidelines.

Fife et al., Neurology, 2008^18^ *American Academy of Neurology (AAN)*	Bhattacharyya et al., 2008^19^ Otolaryngology - Head and Neck Surgery (AAO-HNS).
1 Which maneuvers effectively treat BPPV? 2 Which maneuvers were efficient to manage anterior and horizontal canal BPPV? 3 Were post-maneuver restrictions necessary? 4 Was the simultaneous mastoid vibration important for the efficacy of the maneuvers? 5 Which is the efficacy of the Brandt-Daroff habituation exercises or of the patients’ self-administered maneuvers? 6 Were the medication efficient to treat BPPV? 7 Was surgical occlusion of the posterior canal or singular neurectomy effective to treat BPPV?	1a Posterior canal BPPV; 1b Lateral canal BPPV diagnosis; 2a BPPV differential diagnosis; 2b Modifying factors; 3a Radiograph and vestibular tests; 3b Audiometric tests; 4a Repositioning maneuver as initial treatment; 4b. Vestibular rehabilitation as initial therapy; 4c Observation as initial therapy; 5 Drug therapy; 6a Treatment response reassessment; 6b Treatment failure assessment; 7 Education.

The authors summarized the recommendations presented by each paper. We can identify some common topics between the two files, which will serve as basis for the discussion. (Table 7).

**Table 7:** Comparing the results found in the guidelines.

	Fife et al., Neurology, 2008^18^ (AAN)	Bhattacharyya et al., 2008^19^ (AAO-HNS)
Canalith repositioning maneuver	Level A	Recommendation
Semont maneuver	Level C	No recommendations
Horizontal canal BPPV treatment	Level U	No recommendations
Self-treatment	Level U	No recommendations
Restrictions on post-maneuver activities	Level U	No recommendations
Medication use	Level U	Recommendation against
Vestibular rehabilitation	Level C	Option

**Table 2:** Study Methodology - American Academy of Neurology (AAN)

Fife et al., Neurology, 2008^18^
*American Academy of Neurology (AAN)* The following databases were studied: MEDLINE, EMBASE and Current Contents, concerning relevant papers, published entirely and assessed in pairs, between 1966 and June of 2006. At least two members of the group commented each paper for inclusion. The literature was limited to human beings, randomized and controlled clinical trials, case-controlled and cohort studies, series involving more than six individuals and metanalysis. Summaries, abstracts and papers, with or without improvement statements were taken off the study. The papers included in this analysis met the following criteria: 1) BPPV diagnosed by both symptoms of positional vertigo, lasting less than 60 seconds and paroxysmal positional nystagmus in response to the Dix-Hallpike maneuver or some other provocative adequate maneuver. 2) For all forms of BPPV, nystagmus was characterized by a short latency before nystagmus onset or by a nystagmus reduction with repeated Dix-Hallpike maneuvers (fatigability). 3) For posterior canal BPPV, a positive Dix-Hallpike maneuver was defined by the presence of torsional nystagmus beating upwards, with the upper pole beating towards the affected ear. 4) For posterior canal BPPV, the Dix-Hallpike maneuver or the roll test produced horizontal positional paroxysmal nystagmus changing directions - geotropic (towards the ground) and apogeotropic (opposite to the ground). Geotropic positional nystagmus is associated with the paroxysmal nystagmus beating to the right when the supine head is turned to the right and the paroxysmal nystagmus beating to the left when the supine head is turned to the left, and the opposite happens with the apogeotropic.

**Table 3:** Methodology - American Academy of Otolaryngology (AAO-HNS)^19^

Bhattacharyya et al., 2008^19^
*American Academy of Otolaryngology (AAO-HNS)* Many searches were made, from December of 2007 through February of 2008, in MEDLINE, by AAO-HNS employees, with the following key-words: “BPPV OR Benign Paroxysmal Position Vertigo” or “positional vertigo” or “benign positional vertigo” or “paroxysmal positional vertigo” or “benign paroxysmal positional vertigo”, in the Title or in the Abstract. The papers found followed the following selection criteria: 1 Clinical practice guidelines: “guideline” was searched in MEDLINE, as type of publication or title word, with the topic of vertigo, produced by Medical Association or Organization, and which had an explicit method to obtain evidence and associate them to the recommendations. 2 Systematic review/metanalysis: which contained explicit criteria used to do the bibliographic search and which selected the papers searched by inclusion or exclusion. 3 Randomized and controlled clinical trials: identified in a search in the Cochrane database as controlled trial register, with BPPV in the Title. 4 Original studies: identified limited to a search in MEDLINE for papers with focus on vertigo, published in English, with human subjects and which were not of the case-report type. A guided search was made in order to refine the search pattern. The final summary went through an extensive and careful review from the external observer, and the comments were revised by the head of the group. The recommendations within were based on the best data published by March of 2008. Should the data not be enough, a combination of clinical experience and a consensus among specialists was used.

## DISCUSSION

### BPPV diagnosis

The diagnosis of this condition must be based on clinical history and physical exam and, usually, there are no auditory complaints^20^. The typical story is characterized by vertigo spells upon changes in head position, as the person rolls over to one of the sides in bed, as the person gets up, looks up, bends down, and it may be accompanied or not by nausea or vomit. We may also find instability and unbalance. Symptoms tend to resolve spontaneously within some weeks or months, there may or may not be recurrences. The vestibular system involvement must be checked by means of a neurotology assessment, which may include the search for vertigo and positional nystagmus, spontaneous and semi-spontaneous nystagmus, saccadic eye movements, pendular tracking, head selfrotation, caloric test, and others. Positioning nystagmus can be identified with the use of Frenzel goggles or VNG (videonystagmography), enabling the recognition of the canal involved and ruling out the ocular fixation inhibition effect on the vertical and horizontal nystagmus^20^, ^21^.

In executing the Dix-Hallpike maneuver, the patient is initially seating down, with the head turned laterally in approximately 45°, right or left, according to the side to be tested. With the examiner hold the patient's head, the patient is instructed to lay down in dorsal decubitus. The head remains pending, at an approximately 30°extension. The patient remains immobilized in this position, with the eyes open and the diagnostic criterion includes the occurrence of a characteristic mixed-torsional and vertical nystagmus with the upper eye pole beating towards the dependent ear and upwards, when the posterior semicircular canal is affected. The nystagmus has a 1 to 5 second latency time in cases of canalolithiasis, and between 10 and 20 seconds, in cases of cupulolithiasis. As the patient returns to the seating position, the nystagmus may occur in the opposite direction, with or without vertigo, making up a torsional-type of nystagmus beating downwards. In the modified Dix test, the patient seats on the bed, with his legs hanging out, the head is turned in 45° to one of the sides and lies down on the opposite side. The same Dix Hallpike responses are expected should the patient have BPPV, both for the anterior as well as for the posterior canal. The patient then returns to the seating position, to check whether there is nystagmus in this position, and then the test is repeated on the opposite side^22^.

The roll test is used for the horizontal canal BPPV, in which the patient lies down in dorsal decubitus, with the head flexed anteriorly in 30°. The patient then turns his head to one side and keeps it in this position for up to one minute. A horizontal nystagmus, of lower latency and less prone to fatigue - because the otoconia move inside the canal - is expected. In horizontal canalolithiasis, nystagmus is geotropic or it beats towards the lower portion of the ear, with the fast phase towards the center of the earth, it is fatigable and lasts for less than 60 seconds. While in cupulolithiasis, it is apogeotropic, or towards the upper ear and persistent. In canalolithiasis, the direction of the greatest intensity of this nystagmus usually identifies the affected side.

Some patients who do not have the characteristic nystagmus in the Dix Hallpike maneuver, but experience the classic vertigo during the test will be classified as subjective BPPV and treatable by the maneuver^23^. Bhattacharyya et al.^19^ commented that some factors, such as the speed of the movement, the time of the day and the angle of the occipital plane during the maneuver can influence this test, and they also found differences in efficacy because of differences concerning the maneuvers employed by specialists and non-specialists. After checking class B studies, with a few limitations, they concluded that the diagnostic Dix-Hallpike maneuver is classified as “strongly recommended”, and it must be used by the clinicians, unless there is some clear and compelling logic behind an alternative approach.

### Canal Repositioning Maneuver

In 1992, Epley described the Canalith Repositioning Maneuver (CRM), and this technique used cranial vibration besides a pre-maneuver medication and made the patient's head go through five different positions which enabled the calcium carbonate crystals to move, under the influence of gravity, from the posterior canal to the utricle. Today, most neurotologists and physical therapists use a modified version of this procedure. In this maneuver, the patient leaves the seating down position, moved to a Dix-Hallpike position with the head pending to the side of the affected ear, where it is kept for 30 to 60 seconds. The head is then turned 90° to the opposite Dix-Hallpike position, keeping neck extension. Following that, the patient continues the movement 90° further, until the head is diagonally opposite to the first Dix-Hallpike position, where it is kept for 30 - 60 seconds more. After this position, the patient is sat^23^. Herdman & Tusa^24^ reported a certain controversy concerning the treatment of canalith repositioning, since the studies evaluated, despite boasting rates of 85% to 95% of symptom remission, did not use the control group and discussed the possibility of spontaneous recovery remission.

Bhattacharyya et al.^19^ stated that the posterior canal BPPV must be treated with the Canalith Repositioning Maneuver, based on randomized clinical trials (Class B studies), and small samples, where there was a predominance of benefits over the risks, which classified it as “recommended”. This means that health care professionals must be attentive to new information and must be sensitive to the patient's preference. In their search, the authors found a systematic review, based on three randomized, controlled, high-quality clinical trials, and noticed significant effects favoring Canalith Repositioning (CR) when compared to the control groups (*odds ratio* of 4.2% favoring CR for the subjective resolution of symptoms and a 5.1% odds ratio favoring treatment to convert a positive Dix-Hallpike test into negative). Positive results for CR treatment were also shown in seven non-randomized clinical trials (of lower quality) and case series. At last, four metanalysis concluded that CR is significantly more effective than placebo^19^. Based on two class I studies, three class II, four metanalysis and one systematic review, Fife et al.^18^ classified the maneuver as “Level A Recommendation”, in other words, treatment is effective and safe, and it should be offered to the patients of all ages with posterior canal BPPV.

Korn et al.^25^ and Dorigueto et al.^26^ studied the number of maneuvers which must be used to treat BPPV and concluded that repeated maneuvers in the same session seem to be more efficient, and even more necessary when one is dealing with cupulolithiasis. It was only in one of the studies^19^ hereby analyzed, that it was not possible to identify a specific number or a protocol for such, and the CR repetition must be determined by symptom severity, if persistent, by the professional's evaluation and its successful treatment with the maneuver.

### Semont's Maneuver

In order to treat posterior canal cupulolithiasis, in 1988 Semont described the freeing maneuver, in which the patient starts seating down, with the head in rotation to the healthy side, until he is laid down towards the affected side with the head turned upwards. After 1 to 2 minutes, the patient is quickly moved, going through the seated position, to lie down on the opposite side, with the head now pointing downwards, where the patient remains for 1 to 2 minutes. Afterwards, the patient returns slowly with the head still tilted and fixed until the seated position. It is believed that sudden changes in head position may release the crystals which were adhered to the cupula^27^. Maia et al.^28^ stated that some authors consider the Semont maneuver too aggressive, because it often times triggers severe dizziness and it is not well tolerate by the patients. Contrary to that, Reis^29^ stated that this can be the only solution for the most difficult cases. Both international guidelines hereby discussed concluded that there is no significant evidence to establish Semont's maneuver efficacy in relation to Canalith Repositioning, based on the analysis of the three studies. One Class II study showed that there is a significant improvement (*p* < 0.009) in vertigo intensity for those patients treated in comparison to placebo treatment; the other, class III, we noticed a greater improvement in the use of medication; and, at last, as we compare the Semont's maneuver with the Canalith Repositioning Maneuver and the Brandt-Daroff habituation exercises, in a class III study, the two maneuvers had an effect which was very much similar in the short run (71%, 74% and 24%, respectively); however, CR stood out in the long run (77%, 93% and 62%, respectively)^18^, ^19^. Fife et al.^18^ then stated that the Semont maneuver can be “possibly effective”, a concept based on a single Class II study, resulting in a “Level C Recommendation “. Bhattacharyya et al.^19^ ratified that it is less effective than no treatment or that the Brandt-Daroff exercises in the control of symptoms.

### Horizontal canal BPPV treatment

When approaching horizontal canal BPPV, canalith repositioning and the modified repositioning maneuver are usually inefficient; therefore, some alternative maneuvers have been proposed^18^. Based on 10 and in 2 papers, Fife et al.^18^ and Bhattacharyya et al.^19^, respectively, stated that the roll maneuver (Lempert or *Barbecue*) and its variations are the most commonly employed approaches. A variation would be to modify the original Epley Maneuver, moving the head in a horizontal canal plane, proposed by Herdman and Tusa^24^. The patient in dorsal decubitus with the involved ear pointing downwards, would move his head slowly until reaching the position with the face turned upwards, keeping it like this until the vertigo recedes. The patient continues with the 90° movement to the opposite side to the lesion until completing a 360 degree turn, waiting in each position until the dizziness is gone. The simplest approach is the “prolonged position maneuver”, developed by Vannucchi et al., which is based on the patient's remaining on healthy lateral decubitus for 8 consecutive hours. Libonati mentioned other maneuvers, such as the Vannucchi-Asprella and the Lempert (*barbecue*) maneuver for canalolithiasis (geotropic nystagmus) and the Gufoni maneuver, in the most severe cases of the cupulolithiasis form (apogeotropic nystagmus)^27^_._ Fife et al.^18^ found two class IV studies, with different parameters, not very clear and without a control group to compare it with the rate of natural recovery over this condition. The authors reported that the Lempert's maneuver efficacy is about 75%, varying between 50% and almost 100%. The same result can be seen in the paper published by Bhattacharyya et al.^19^, based on fifteen Class C papers. The Gufoni's and Vannucchi Asprella's maneuvers were checked by Fife et al.^18^ and considered effective; however, there are only data from Class IV studies (four involving the first one and three limited papers supporting the latter). The prolonged forced position, presented by approximately the same number of papers, was also reported as effective in both guidelines^18^, ^19^. The same maneuvers, as assessed by Bhattacharyya et al., based on three non-controlled studies, were also reported as effective. The authors concluded that there was not sufficient evidence to recommend it as a preferable treatment for horizontal canal BPPV^19^.

### BPPV self-treatment

According to Bhattacharyya et al.^19^, no comparative and carefully conducted study was published in order to make recommendations concerning self-treatment versus a treatment given by a professional. They believe that, in motivated individuals, BPPV self-treatment may be an option. Fife et al.^18^ classified it as a Level U Recommendation”, because, there was no sufficient evidence to recommend or refuse self-treatment using the Semont or Canalith Repositioning maneuvers. We found three papers. One of them reported a mild improvement in those patients who were instructed to self-administer CR at home after starting it in the medical office (88% compared to 69%, when done only at the office). The remaining two compared the self-administered Canalith Repositioning Maneuver with the Brandt-Daroff exercises and with the self-administered Semont Maneuver. The results were 64% and 95%, respectively, of improvement with the repositioning, compared to 23% with the Brandt-Daroff exercises and 58% after self-administration of the Semont maneauver^18^, ^19^_._

### Post-maneuver activity restrictions

Some controversies in the literature are found when one is dealing with the efficacy of imposing restriction of some activities to the patients after the canalith repositioning maneauver^28^, ^29^, ^30^, ^31^. Once again, both authors of the guidelines did not find evidence to corroborate this efficacy^18^, ^19^. Upon examining 6 class IV studies, Fife et al.^18^ classified the restrictions as “Level U Recommendation”, since five of the six studies did not show any additional benefit of the post-maneuver restrictions, and only one showed minimum benefit in patients with such restrictions, as measured by the number of maneuvers necessary to produce a negative Dix-Hallpike test.

### Medication

Ganança et al.^32^ advocated that the use of a combined treatment modality can lead to quicker and more long lasting symptom improvement or resolution than monotheratpy, establishing that betahistine, cinnarizine, clonazepam, flunarizine or Gingko Biloba extract ameliorate vestibular vertigo. On the other hand, Konnur^33^ believes that through medication one can get to a satisfactory symptom improvement during acute crises, but they can be potentially counter-producing as to central vestibular compensation, especially if used for longer periods of time. Medication usually employed to manage acute symptoms are: anti-dizziness agents, anti-histaminic or vasodilators; and these may cause sedation and central nervous function depression. As symptoms subside, the drugs must be discontinued and the patient should start with vestibular rehabilitation. According to the hereby compared guidelines, the recommendations were: “recommendation against”, or “level U recommendation”. Fife et al.^18^ concluded that no evidence was found to support the recommendation of any medication in the routine treatment of BPPV. They got to this conclusion after assessing two class III studies. The first experiment did not find any difference between lorazepam (1mg, TID), diazepam (5mg, TID) and placebo, after four weeks of treatment. In the second one, flunarizine proved to be more effective than not treatment at all and less effective than the Semont Maneuver in eliminating the symptoms^18^.

Bhattacharyya et al.^19^ treated the matter more in depth, analyzing a larger number of papers and they also did not find evidence to suggest that some vestibular suppressive agent could be effective as a primary and definitive treatment for BPPV, or a substitute for repositioning maneauvers^19^. Some studies showed BPPV resolution in the long run with the use of medication, but these did not follow the patients up for a period of time in which spontaneous remission could occur. A small study compared CR with medication monotheratpy and concluded that canalith repositioning yielded substantially better responses (78.6% to 93.3%, respectively) when compared to the use of medication alone (30.8% of improvement), after two weeks of follow up. The lack of vestibular suppression benefits and its inferiority to CR indicate that clinicians should not avoid the maneuver and prefer medication to treat BPPV, having seen that some drugs may yield side effects which interfere in vestibular compensation, they mask findings from the Dix-Hallpike maneuver, they interfere in the cognitive function, gastrointestinal mobility, they impair urinary function and cause movement and vision disorders^19^. Thus, international guidelines point out that the vestibular function suppression agents are not recommended for the treatment of BPPV, except for the short term management of neurovegetative symptoms such as nausea and vomit in a severely symptomatic patient, and for patients who became severely symptomatic after a CR. These conclusions made by Bhattacharyya et al.^19^, were obtained from a class C, observational and crosssectional study.

### Vestibular rehabilitation

During the 40's, physician Cooksey and the physical therapist Cawthorne, proposed the use of exercises - vestibular rehabilitation - with the goal of treating vestibular disorders. The program was based on a series of eye, head and body movements, usually in the positions in which the rotational dizziness was triggered and which should be done according to the patient's tolerance and his individual needs. Considered as a therapeutic approach, vestibular exercises try to bring about an improvement in global balance, quality of life, restoring special orientation to as close as possible to physiological conditions. This recovery happens by means of vestibular compensation. Besides these mechanisms, there may also be adaptation, habituation and substitution^33^. As far as adaptation is concerned, the vestibular system can learn again to receive and process information, even when distorted or incomplete, adapting itself to the stimuli presented in order to recover from the altered reflex. Habitation tackles the symptoms, being based on the reduction of sensorial responses based on the repetition of sensorial stimuli, made possible by the repetition of movements, causing a reduction in vestibular response and reduction in nystagmus amplitude. For that, it is necessary to integrate all involved sensorial inputs: visual, vestibular and somatosensory. In the vestibular substitution process there is an exchange of information associated with body balance which are absent or conflicting. Later modified by Brandt and Daroff, habituation exercises require that the patient move in the triggering position, repeatedly and many times during the day. The Brandt-Daroff exercises are usually indicated in cases of less intense BPPV, as a coadjuvant to the Epley and Semont maneauvers^34^. During its execution, the patient is seating down and turning his head in up to 45° towards the side which does not cause vertigo, and lies down towards the side that causes the symptoms, remaining in this position for 30 seconds, or until vertigo ends; after this time, the patient should seat again, for 30 seconds. Following that, the patient lies down again to the opposite side and remains there for 30 seconds more, until returning to the seated position. The exercise duration and frequency depend on neurotological findings and patient evolution, and it should be customized for each case and repetition many times per day is indicated until positioning vertigo subsides for at least two consecutive days^34^, ^35^, ^36^. Fife et al.^18^ evaluated only two studies without getting to any specific conclusion concerning the use of vestibular rehabilitation exercises^18^, while Bhattacharyya et al.^19^ described many papers and, based on class C, observational, limited, controlled and randomized trials, listed such exercises as optional for the treatment of BPPV. They then concluded that, regarding posterior canal BPPV, that vestibular rehabilitation yielded better results for BPPV treatment when compared to placebo. In the short term assessment, the exercises proved to be less efficient in producing complete symptom resolution when compared to CRM. Nonetheless, with long term follow up, its efficacy comes close to that of the repositioning maneuver. Thus, vestibular rehabilitation is considered possibly better indicated as an adjuvant treatment instead of being a primary treatment modality.

## CONCLUSION

After considering the treatment proposals for each guidelines we may conclude that the Dix-Hallpike maneuver was considered a gold standard for the diagnosis of BPPV. As far as treatment is concerned, we noticed that the only one with sufficient recommendation was the canalith repositioning maneuver, which is the best option to treat vertical canalolithiasis and the one with the most high quality publications advocating it. The Semont maneuver is possibly efficient, but there is still the need to develop better studies concerning this technique. Regarding self-administered treatment and post-maneuver activity restrictions, there were no sufficient studies to support its recommendation. As to the intervention with multiple maneuvers, it was not possible to find a specific number or protocol which could justify its efficacy. CRM repetition must be determined by symptom severity, should they persist, by the expert's assessment and the past success the expert had with the procedure. Medication is contra-indicated, but there are not enough papers supporting such statement. Vestibular rehabilitation is considered possibly efficient, thus being a secondary option to treat BPPV. Of the studies hereby discussed, the work led by Bhattacharyya et. al and under the auspices of the American Academy of Otorhinolaryngology seems to be the most comprehensive and in-depth concerning BPPV. Besides having a more complete team of researchers and clinicians, they had the largest number of publications, which provided them with greater scientific basis to recommend each technique. We also found in them, not only a detailed analysis of the data obtained, but also a care with the introduction of each topic discussed. The study carried out by Fife et al.^18^ was considered more limited, since it was restricted to respond only those questions made and they had a limited team. The small number of references may be associated with the study limitation. Despite this difference in its creation and in some guidelines’ content hereby discussed, both are qualified studies, highly recommended for health-care professionals who wish to add more to their knowledge about BPPV diagnosis and control.
